# Acute Pyelonephritis in Pregnancy: A Retrospective Descriptive Hospital Based-Study

**DOI:** 10.5402/2012/519321

**Published:** 2012-11-14

**Authors:** J. C. Dawkins, H. M. Fletcher, C. A. Rattray, M. Reid, G. Gordon-Strachan

**Affiliations:** ^1^Department of Obstetrics and Gynaecology, University of the West Indies, Mona, Kingston 7, Jamaica; ^2^Tropical Metabolism Research Unit, University of the West Indies, Mona, Kingston 7, Jamaica; ^3^Dean's Office, Faculty of Medical Sciences, University of the West Indies, Mona, Kingston 7, Jamaica

## Abstract

*Introduction*. Pyelonephritis is a common complication of pregnancy. It is also exacerbated by immunocompromised states and also the sickle cell gene. We reviewed this condition in Jamaican women. *Method*. We did a six year hospital database docket review. We found 102 confirmed cases. *Results*. Pyelonephritis was found in 0.7% of deliveries. The mean maternal age was 24 ± 5.83 years with 51% primiparity. Most (58.8%) occurred in the second trimester. The main symptoms were loin pain (96.2%) and abdominal pain (84.6%). It was more common on the right side in 67% of cases. On urinalysis, 81.4% had pyuria. The commonest organism was *Escherichia coli*, in 61% of cases. Patients given Antibiotics prior to admission had quicker resolution, *P* < 0.02. Haemoglobin S was found in 16% cases (general population 10%; *P* = 0.002). However diabetes was only found in 1.3% cases (1.5% expected). 61.3% had positive urine culture after treatment showed that 61.3% and 25% had recurrent pyelonephritis. Complications included 32% threatened preterm labour and 17% preterm delivery. About 6% of neonates had intrauterine growth restriction. There were no ICU admissions and no deaths. *Conclusion*. Early recognition and treatment of pyelonephritis result in good outcome. The condition is more prevalent in patients with the sickle cell gene and recurrence is high.

## 1. Introduction

Acute pyelonephritis complicates approximately 1-2% of pregnancies, and is one of the leading causes of nonobstetric antepartum hospitalization [1, 2]. Earlier epidemiological data showed an incidence as high as 10% [3], but with improved antenatal surveillance, the incidence of acute pyelonephritis has decreased in recent years. However, the development of antibiotic resistance and other factors have now affected the diagnosis, clinical course, and treatment.

The incidence of asymptomatic urinary tract infection does not increase in pregnancy compared to the non-pregnant state [4], but physiological and anatomical changes causing urinary stasis in pregnancy increases the risk of clinical disease [5], especially in mid pregnancy [6] and more commonly on the right side [7]. African American multiparous women with the sickle cell trait have been found to have the highest incidence of asymptomatic bacteriuria (ASB) [8]. Screening and treatment of ASB, found in 6% of gravidas [9], have been shown to reduce pyelonephritis risk by 70 to 80% [10]. At the University Hospital of The West Indies (UHWI), urine culture screening is not done, but dip stick testing is performed at each visit. 

In pyelonephritis, infection usually ascends from the bladder, due to vesicoureteral reflux in pregnancy [8]. Other risk factors include previous episodes of pyelonephritis, abnormalities or stones of the urinary tract, and other conditions such as diabetes mellitus, sickle cell disease, and AIDS. 

Several studies have demonstrated maternal and fetal morbidity [1, 11] associated with pyelonephritis. Fever in the first trimester during organogenesis has been associated with teratogenicity, miscarriage, and preterm delivery. Increased Uterine activity occurs due to the presence of endotoxin [12] and also due to the fever [11]. The presence of endotoxin is also said to cause hemolytic anaemia in one third of these patients [13].

Management of suspected urinary tract infections at UHWI is very aggressive. The presence of leukocytes or persistent proteinuria on dip stick testing mandates that a midstream urine sample (MSU) be sent for culture and sensitivity, with treatment based on the results. Women with abdominal discomfort suspicious of UTI and all women with preterm labor have MSU testing and are started on empirical antibiotics. All suspected cases of pyelonephritis are hospitalized and treated with intravenous antibiotics until afebrile for 24 hours and symptomatically improved [14]. This aggressive approach has been employed to minimize the possibility of renal damage. Newer diagnostic techniques, the acceptance of short-course therapy for lower tract infections, and the development of new antibiotics have increased the diagnostic and therapeutic options by others [15].

In Jamaica, the prevalence of Type 2 diabetes, in adult women is 15.7% [16]and the prevalence of sickle cell S trait is 10% [17]. There is consensus that ASB and pyelonephritis are more prevalent in diabetes mellitus [18] and is associated with high proportion recolonization after antibiotic treatment for ASB [19] in pregnancy; diabetes is an independent risk factor for ASB [20]. The presence of the HB S gene is associated with increased risk of pyelonephritis in pregnancy [21–23]. Therefore, in the Jamaican context we hypothesized that a significantly higher proportion of women with pyelonephritis in pregnancy should have diabetes or the Hb S gene.

The maternal and foetal outcomes as well as the determinants of pyelonephritis in pregnancy at the UHWI are unknown. This study was therefore done to evaluate these.

## 2. Materials and Methods

The study was retrospective, using the UHWI Hospital database from January 2003 through December 2008 using a diagnosis of acute pyelonephritis in pregnancy. The dockets were retrieved and reviewed and those who met the clinical or laboratory criteria for the diagnosis of acute pyelonephritis were included. 

The diagnostic criteria of acute pyelonephritis employed were flank pain, nausea/vomiting, fever (>38°C) before or on admission, and/or costovertebral angle tenderness, in the presence or absence of cystitis symptoms. A positive urine culture was defined as >10^5^ CFU per mL of organisms. For patients who had multiple admissions, the information obtained was for the first admission. 

Demographic data collected were age, weight, body mass index, gravidity, and parity. The gestational age of diagnosis was recorded. Medical history deemed to place patients at risk for pyelonephritis was also collected. Outcome analyzed were length of hospital stay, time to defervesce, organisms involved and the sensitivity to antibiotics, presence of preterm labour and premature rupture of membranes, interval between infection and delivery, Apgar scores, and birth weight.

The study was approved by the ethics committee of the University of the West Indies.

## 3. Statistical Analysis

Values are presented as means with standard deviation (SD), medians with interquartile ranges or counts as appropriate. For continuous outcome variables with normal distribution differences in means by categories were tested with analysis of variance (ANOVA) or *t* test as appropriate. For continuous outcome variables that were skewed, differences in distribution by categories were tested with Kruskal-Wallis test or the Mann-Whitney test.

For categorical outcome variables, differences were tested with chi square. The binomial test was used to compare the observed proportion in this study with expected proportion in the general population.

## 4. Results

A total of 282 patients were coded as pyelonephritis. We recovered 184 dockets (65.2%). Of these, acute pyelonephritis was confirmed in 102 (55.4%). During the time period there were a total of 14651 deliveries, giving an incidence of 0.71% of deliveries. 

The mean maternal age at diagnosis was 24 ± 5.83 years (mean ± SD), with the majority of cases (62 of 102 patients or 61%) within the age range of 20–29 years. There were 8 patients (8%) who were older than age 35 years (Table 1). About a half of the women (51%) were nulliparous.

The majority of cases of acute pyelonephritis (58.8%) occurred in the second trimester, followed by third trimester (28.5%) and first trimester (14.7%). The mean gestational age at diagnosis was 22 ± 7.8 weeks (Table 1). 

All patients were admitted for treatment and received intravenous fluid and antibiotics on admission. The commonest complaints on admission were loin pain (96.2%), abdominal pain (84.6%), dysuria (70.2%), fever (64.4%), and chills and rigors (51.9%). Twenty three percent of patients gave a history of antibiotic use in the week prior to admission. Fever defined as temperature >38°C on admission was less common in patients who gave a history of antibiotic use in the week prior to admission (*χ* ^2^ = 5.58, df(1), *P* < 0.02).

On urinalysis, 81.4% of cases had pyuria, 29.4% were nitrite positive, and 38.2% had microscopic hematuria.

The mean haemoglobin at admission was 10.4 g/dL. 32 patients (30.7%) had haemoglobin of less than 10 g/dL at admission and of these, 5 had sickle cell disease. 20 patients (19.2%) had absolute white blood cell count (WBC) greater than 15.0. The median WBC was 12.1 × 10^9^/L with a range of 4.5–23.3 × 10^9^/L. There were 17 cases (16.3%) with electrolyte derangements, but no patients required intensive care admission.The mean body temperature at admission was 37.8 ± 1.0 degrees Celsius and renal angle tenderness was more common on the right side in 68 of 102 cases (67%) than left side (19/102, 19%), and was bilateral in 14 cases (14%) Table 1.

Fifty-nine cases (56.7%) had positive urine culture, whereas 11 (10.6%) had no growth. There were 15 cases each (14.4%) of insignificant mixed growth and gross contamination. Four subjects had no data regarding urine culture. The predominating organism was *Escherichia coli*, accounting for approximately 61% (36 of 59) of cases. The other major organisms were *Klebsiella* spp. (15.2%, nine of 59) and *Proteus* spp. (5.1% three of 59). *E. coli* was most sensitive to intravenous Amoxicillin-Clavulanate in 88.8% and Gentamycin in 83.3%. Oral Amoxicillin-Clavulanate was used most commonly in 76.3%, followed by oral Cefuroxime in 17.9% of cases. Other antibiotics used were Cotrimoxazole and the urinary antiseptic Nitrofurantoin. Six patients were placed on Nitrofurantoin (as long term prophylaxis) for the remainder of the pregnancy.

The median duration of intravenous antibiotics was three days (range 1–10 days), and seven days (range 1–14) for oral antibiotics. 

The majority of patients (42/62 67.7%) became afebrile within 48 hours of admission. There was no statistical difference in the number of days (median with interquartile range) admitted by trimester. These were 5 (IQR2) days, 5 (IQR 2.5) days, and 4 (IQR2) days for the first, second, and third trimesters, respectively. The number of days to become afebrile was significantly shorter in the women who were treated with antibiotics prior to admission (1.5 ± 1.3 versus 0.75 ± 1.1; *P* < 0.02) (Table 2).

Sickle cell status was available for 73 patients. Of these, 57 (78.1%) were negative, 11 (15.1%) had the trait, and 5 (6.8%) had sickle cell disease.

The observed proportion 16% was significantly greater than the general population proportion of 10% (two-tail binomial test; *P* = 0.002) Patients who were sickle negative spent an average of 4.8 ± 1.7 days compared with patients with sickle cell disease who were admitted for 6.4 ± 1.14 days *P* = 0.14. This was however not statistically significant (Table 3).

Data on diabetes status was recorded in 79 women. Of these only 1 person (1.3%) had gestational diabetes. 

Seventy patients (68.6%) had obstetric ultrasound at admission, and all these were normal. Twenty patients had urinary tract ultrasonography done during admission, 6 of these were normal, 10 (50%) revealed hydronephrosis, and 4 (20%) revealed ureteric stones. 

Seventy patients (68.6%) had repeat urine culture done after completion of the antibiotic course. Of these, 43 specimens (61.3%) were positive and 25 patients (24.0%) were readmitted for recurrent pyelonephritis. 

Of 102 patients diagnosed and admitted with pyelonephritis, 68 (65.4%) were patients of the antenatal clinic with complete antenatal records including delivery outcome. In 36 (34.6%) patients delivery outcome was unavailable for outcome analysis.

Of the 68 cases that were delivered at UHWI, 22 (32%) had threatened preterm labour, 4 (3.9%) had preterm labour, 6 (8.8%) had premature rupture of membranes. Four infants (5.9%) were found to have intrauterine growth restriction on ultrasound. 

Twelve (17.6%) subjects delivered prematurely despite the use of tocolytics and 10 infants (14.7%) were found to have low birth weight. The mean infection to delivery interval was 103 days (SD 50.6) median 110 days range 3–217 IQR 72 days. The median gestational age at delivery was 39.0 weeks, with a range of 31.7–41.0 weeks. The gestational age at delivery was statistically less in patients who had acquired the infection in the first trimester (*P* = 0.03). The median birth weight was 3.14 kg, with a range of 1.67–4.36 kg. The Apgar scores were within normal range with no statistical difference demonstrated by trimester (Table 4).

## 5. Discussion

The incidence of obstetric pyelonephritis at UHWI was 0.7%, slightly lower than reported by others, 1.3–2% [24, 25]. It was seen most commonly within the age group of 20–29 years, with a mean age of24 ± 5.83 in keeping with the age range of patients seen in this clinic. A half of patients were nulliparous, lower than in previous studies where 75% of the cases were nulliparous [25].

The majority of patients demonstrated pyuria on urine dipstick, but less than a third demonstrated positive for leucocyte esterase nitrite, which is said to be a useful cost effective screening test when the prevalence of pyelonephritis is 2% or less as is the case here [26]. Previous studies had demonstrated higher values, but concluded a low sensitivity for this test [27]. 

Anaemia was found in 30% of cases exactly the same as reported by Cox et al. [13]. The data set however did not allow us to determine the type of anemia in this cohort. However clinicians should be mindful of the complication of hemolytic anemia especially in women not responding to hematinics.

The majority of patients became symptomatic in the second trimester. This is consistent with the peak period of urinary stasis and maximal immunological changes on the urinary tract under hormonal influence [6, 28]. Additionally, acute pyelonephritis was more common on the right side, also in keeping with more marked physiological changes on that side [6, 29]. 

The predominant organism isolated was *Escherichia coli*, consistent with previous international studies [7, 30] with 88% sensitivity to Amoxicillin/Clavulanic acid. This is available in parenteral and oral form, granting this population excellent antimicrobial coverage pending even on discharge home. Most patients became afebrile within 24–36 hours of appropriate antibiotics, a finding consistent with previous studies [28, 31]. The average duration of admission was 5 days ±1.9, comparable to findings reported by Sharma and Thapa [24].

Previous studies have demonstrated an incidence of acute pyelonephritis in pregnant sickle cell trait patients that was significantly higher than that in matched control patients [21]. Our population has an approximately 10% prevalence of sickle cell trait, and 15% of those tested had sickle trait, and 6.8% had sickle cell disease. 

Diabetes mellitus has been shown to be a risk factor for acute pyelonephritis [18, 19]. There was only one patient in the study with pre-gestational diabetes mellitus this was significantly lower than the 15% found in the population, as the pregnant women were young women. In fact gestational diabetes has been found in only 1.5% such women [32]. The absence of HIV in the cohort is easier to explain since this is not very common in this clinic incidence of about 0.5% [33].

Ideally, repeat urine culture after treatment is important to identify patients at risk of recurrence [2]. In other countries, the risk of recurrence has been reported to occur in one third of cases [34]. This study demonstrated a slightly lower recurrence rate of 24%. The high incidence of persistent infection (61%) demands close surveillance of these women and also in some cases urinary antiseptics for the rest of the pregnancy as was done in 6% of the women. 

The presence of urinary tract abnormalities is also possible in some of these women and should be sought in those not responding to treatment [8]. Nephrolithiasis was found in 4 patients or 1 : 3662 deliveries which was very similar to the 1 : 3300 deliveries found by Butler et al. [35] in a study from the US. However other USA researchers have reported a much higher incidence of urolithiasis 1 : 244 attributed to geographic location and Caucasian ethnicity that are believed to be at greater risk [36].

There was no significant adverse maternal outcome in this study, probably due to aggressive management [7]. The literature has reported an association with preterm delivery and low birth weight with acute pyelonephritis [8]. Even though there was a shorter infection to delivery interval in patients who had acquired the infection in the first trimester, birth weight and Apgar scores were not significantly affected. The low birth weight incidence of 14.7% was identical to that reported by Sharma and Thapa [24] of 14%, however the preterm delivery rate of 17% was twice as high as that of 7.8% also described by the same group. The ten year data for low birth weight and preterm delivery for UHWI 2001–2010 are 13.5% and 7.1%, respectively. While Sharma and Thapa [24] have concluded that these rates are the same as all other births in their hospital, the findings of this study have revealed a significantly higher preterm birth. This is one of the main causes of perinatal mortality at UHWI. So although the prevalence of pyelonephritis is low we would recommend routine booking mid-stream urine test for ASB to prevent this serious problem.

## 6. Summary

Acute pyelonephritis is associated with sickle cell gene and results in preterm delivery. Persistent bacteriuria is very high but severe maternal complications are low in this cohort.

## Figures and Tables

**Table 1:** Demographic data and general findings.

Variable	Mean	SD
AGE at diagnosis (yr)	24.0	5.8
WEIGHT at booking (kg)	66.7	15.1
BMI (kg·m^−2^)	24.9	4.9
Gestational age at diagnosis (weeks)	22.1	7.9
Gestational age at delivery (weeks)	38.4	2.1
Duration of hospitalization	5.0	1.9
Haemoglobin (g/dL)	10.4	1.4
WBC (blood) × 10^6^/L	12.0	4.1
Maternal pulse/min	101	16
Temperature °C	37.8	1.0

**Table 2:** Fever status and prior use of antibiotics.

Fever status on admission	Antibiotic use in the week prior to admission		Total
	No	yes
Afebrile	40	19	59
Fever	37	5	42

Total	77	24	101

*χ* ^2^ = 5.58, df(1), *P* < 0.02.

**Table 3:** Outcome by sickle cell status.

Sickle cell status		Number	Percent	Mean number of days admitted	Standard deviation
Sickle	Negative	57	78.1	4.8	1.7
	Trait	11	15.1	5.0	1.5
	Disease HbSS	2	2.7	6.4	1.14
	HbSC	2	2.7		
	HbSB 0Thal	1	1.4		

	Total	73	100.0		

*P* = 0.14.

**Table 4:** Neonatal outcome by trimester.

Variables	Trimester of acquiring first pyelonephritis: median (range) {IQR}			*P* value
	1	2	3	
Gestational age at delivery (weeks)	36.6 (31.7–39.0)/{3.6}	38.6 (34.0–40.7)/{1.8}	38.5 (32.6–41.0)/{3.0}	0.03
Birth Weight (kg)	2.91 (1.74–3.62)/{1.28}	3.18(2.12–4.08)/{1.96}	3.18(1.67–4.36)/{0.59}	0.87
APGAR at 1 min	8 (7–9)/{2}	9 (8-9)/{1}	9 (8-9)/{1}	0.60
APGAR at 5 mins	8 (7–9)/{2}	9 (7–10)/{3}	9 (8–10)/{2}	0.67
